# A low-cost three-wavelength illumination system for camera-based photoplethysmography imaging

**DOI:** 10.1016/j.ohx.2026.e00787

**Published:** 2026-05-10

**Authors:** Michal Labuda, Ivan Kuchta, Veronika Wohlmuthova, Jan Seleng

**Affiliations:** a University of Zilina, Faculty of Electrical Engineering and Information Technology, Department of Electromagnetic and Biomedical Engineering, Univerzitna 8215/1, Zilina 010 26, Slovakia; b Comenius University in Bratislava, Jessenius Faculty of Medicine in Martin, Institute of Medical Biophysics, Mala hora 4A, Martin 036 01, Slovakia

**Keywords:** Photoplethysmography imaging, 3-λ illumination, Low-cost design, Non-contact biosensing

## Abstract

This paper presents the design and implementation of a low-cost, reliable three-wavelength (3-λ) illumination device intended for photoplethysmography imaging (PPGI) measurements. The proposed system employs high-power light-emitting diodes operating in the green (520 nm), red (625 nm), and near-infrared (855 nm) spectral regions, enabling non-contact cardiovascular monitoring with flexible spectral configuration. The illumination module integrates seven green LEDs, eleven red LEDs, and fifteen infrared LEDs, a distribution selected to compensate for wavelength-dependent camera sensitivity while providing sufficient radiant intensity and spatial uniformity at low power consumption and cost. Each wavelength channel is independently driven using dedicated single-channel linear LED drivers with pulse-width modulation control, allowing precise adjustment of illumination intensity. System operation is managed by a microcontroller-based control platform, which ensures accurate timing, synchronization, and stable operation required for PPGI applications. The compact and modular design supports straightforward integration with camera-based imaging systems, while optional wireless control enhances usability in laboratory environments. Overall, the proposed illumination device represents a robust and cost-effective solution for experimental and applied PPGI measurements in research-oriented non-contact biosensing systems.

## Graphical abstract

**Figure:**
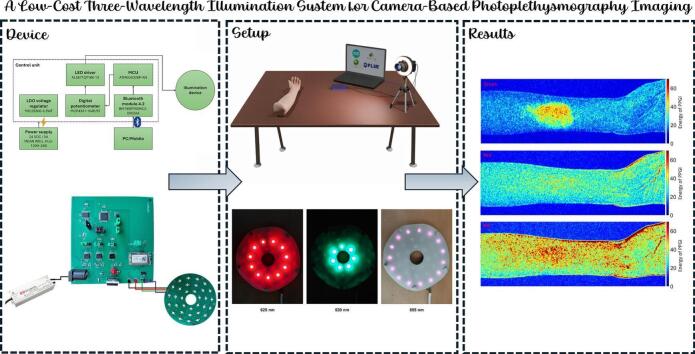

## 1 Specifications table

**Table:** 

Hardware name	A Low-Cost Three-Wavelength Illumination System
Subject area	Educational tools and open source alternatives to existing infrastructure
Hardware type	Electrical engineering and computer science
Closest commercial analog	Multi-Colour Panel – Blue 450 nm – Green 528 nm – Red & Near-Infrared
Open source license	CC BY 4.0
Cost of hardware	105 €
Source file repository	*https://doi.org/10.17632/cj2bwfxrg4.2*

## 2 Hardware in context

Photoplethysmography imaging (PPGI) is a contactless optical technique that enables the extraction of physiological information related to pulsatile blood volume changes by analyzing subtle spatiotemporal variations in skin reflectance (specular and diffuse) captured by cameras. One of the most established applications of PPGI is remote heart rate monitoring, where cardiac pulse signals can be robustly recovered from facial or skin videos under ambient illumination conditions [1], [2]. Beyond heart rate estimation, PPGI has also been successfully applied to the assessment of heart rate variability (HRV), providing a non-contact proxy of autonomic nervous system activity and cardiovascular regulation [3].

PPGI has demonstrated significant potential in respiratory monitoring, as respiration-induced modulations of the pulsatile waveform enable remote estimation of respiratory rate [4]. Additionally, PPGI has been explored for spatial mapping of tissue perfusion and vascular function, including the visualization of heterogeneous blood flow patterns, making it useful in applications such as wound assessment and burn monitoring [5].

In clinical settings, PPGI is particularly advantageous in scenarios where contact-based sensors are impractical, such as neonatal intensive care, burn units, and infectious disease monitoring, where minimizing physical contact reduces the risk of skin injury and cross-contamination [6], [7]. The technique is also increasingly applied in telemedicine and remote patient monitoring, enabling unobtrusive and continuous cardiovascular assessment without wearable devices [8].

Furthermore, PPGI has gained attention in affective computing and mental health applications, including stress detection, emotional state analysis, and cognitive workload assessment, by analyzing pulse dynamics and HRV remotely [3]. In consumer and wellness domains, PPGI is being integrated into fitness tracking, sleep monitoring, and human–computer interaction systems, highlighting its growing relevance beyond traditional medical environments [9]. Overall, the versatility, non-contact nature, and compatibility with low-cost imaging hardware make PPGI a promising technology for future healthcare, telemonitoring, and human-centered sensing applications.

**Fig. 1:**
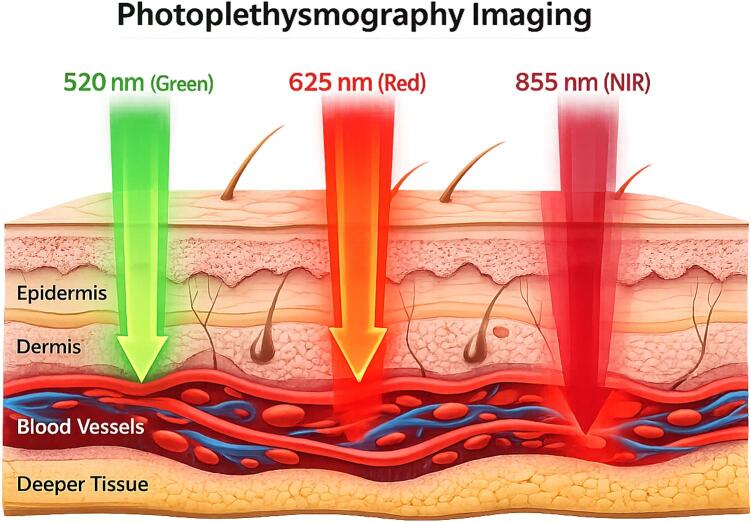
Light penetration through skin.

PPGI detects subtle temporal modulations in diffusely reflected light, resulting from wavelength-dependent absorption and multiple scattering in skin tissue, which are modulated by pulsatile blood volume changes in the microvasculature. The wavelength of illumination critically influences the effective sampling depth, absorption, scattering, and reflectance of light within skin tissue, directly affecting signal quality. Shorter wavelengths, such as green light (525  nm), Fig. 1, experience stronger absorption and scattering in skin, leading to predominantly superficial sensitivity to capillary networks and upper dermal microvasculature [5], [1]. Medium wavelengths, Fig. 1, such as red light (625  nm), probe deeper dermal layers with reduced attenuation, while near-infrared (NIR) light, 855  nm, Fig. 1, exhibits the largest effective penetration depth and reduced sensitivity to epidermal melanin absorption, making measurements more robust across different skin tones [1], [3], [10].

The absorption of light by hemoglobin is wavelength-dependent, with green light absorbed strongly, red light moderately, and NIR minimally [3], [5]. Melanin absorbs strongly in green and red wavelengths but weakly in NIR [3], [11]. By combining multiple wavelengths, PPGI systems can exploit differential absorption and diffuse reflectance from tissue to enhance pulsatile signal detection, improve signal-to-noise ratio, probe complementary vascular compartments at different effective depths [12]. Multispectral illumination also facilitates computational separation of physiological signals from motion- and illumination-related artifacts, increasing robustness and reliability of remote cardiovascular monitoring [7].

## 3 Hardware description

### 3.1 Measuring setup

**Fig. 2:**
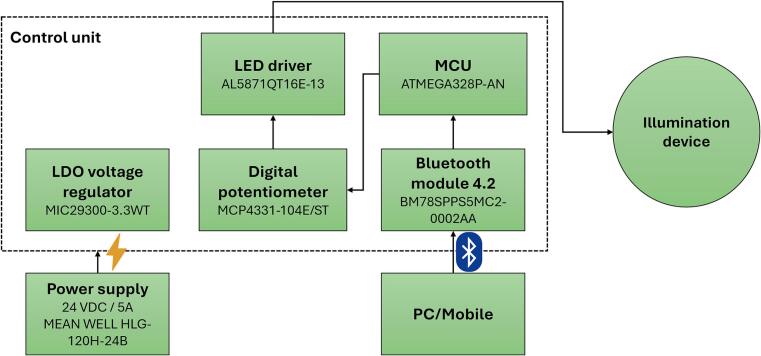
Block diagram of the proposed device.

The primary objective of this work was to design a low-cost, reliable, and configurable illumination system. The proposed device is based on commonly available electronic components and allows flexible control of spectral composition and light intensity. The main hardware components of the system include a microcontroller, a digital potentiometer, LED drivers, a Bluetooth communication module, and a switch-mode power supply, Fig. 2.

The central control unit of the system is the ATmega328P (Microchip Technology, Chandler, USA) microcontroller, which manages the overall operation of the illumination device. The microcontroller configures the operating parameters, including the selection of illumination modes (single-wavelength to three-wavelength operation), the switching frequency of LED chains corresponding to specific wavelengths, and the overall light intensity. The ATmega328P was selected due to its low power consumption, widespread availability, and sufficient peripheral support for embedded control applications [13].

Light intensity control is achieved using a digital potentiometer MCP4331-104E/ST (Microchip Technology, Chandler, USA), which is connected to the LED drivers and operates in rheostat mode. This configuration enables independent adjustment of the current, and therefore the luminous intensity, for each color-specific LED chain. Digital potentiometers provide improved repeatability and remote configurability compared to analog alternatives [14].

The LED driver circuits AL5871QT16E-13 (Diodes Incorporated, Plano, USA) are responsible for switching and regulating the individual LED strings based on control signals from the microcontroller. These drivers ensure stable current regulation and reliable operation of the LED chains, which is essential for maintaining consistent optical output and preventing thermal overstress [15].

The experimental setup utilizes multiple high-power LEDs with different spectral characteristics: 7 × PM2E-3LGE-SD (ProLight Opto, Taiwan), 11 × PM2E-3LRE-SD (ProLight Opto, Taiwan), and 15 × PM2E-2LJE-SD (ProLight Opto, Taiwan) LEDs. This configuration enables the generation of various spectral compositions tailored to specific illumination requirements [16], [17].

Wireless communication is provided by the BM78SPPS5MC2-0002AA (Microchip Technology, Chandler, USA) Bluetooth module, which allows the illumination system to be controlled remotely using a mobile phone or a laptop computer. The module supports Bluetooth Serial Port Profile (SPP), simplifying integration with external control software and enhancing user accessibility [18].

The entire system is powered by a Mean Well HLG-120H-24B (MEAN WELL Enterprises Co., Ltd., Taipei, Taiwan) constant-voltage LED power supply. This industrial-grade power source offers high efficiency, built-in protection features, and long-term reliability, making it suitable for continuous operation in laboratory and industrial environments [19].

The main contributions of this work are:

(1) development of a low-cost multispectral illumination system with independent wavelength control,
(2) integration of synchronized LED switching and camera triggering,
(3) open-source implementation enabling reproducibility and accessibility,(4) comprehensive validation including optical, thermal, and safety characterization.

To justify the designation of the proposed device as a low-cost solution, a comparison with representative PPGI systems is provided in Table 1. In practice, complete PPGI systems consist of an imaging unit (typically an industrial camera), an illumination subsystem, synchronization/control electronics, and a processing unit. Commercial and research-grade implementations commonly rely on high-performance machine vision cameras and dedicated illumination hardware, resulting in total system costs in the order of several thousand euros.

**Table 1:** Cost comparison of representative PPG and PPGI systems and the proposed solution.

System type	Example / configuration	Main functionality	Approximate total cost
Research PPGI system (typical lab setup)	FLIR Blackfly S + LED illumination + PC	Remote multispectral PPGI	2000 – 6000 €
Multi-camera rPPG system	2–4 cameras + GPU workstation	Multi-subject monitoring	3000 – 10,000 €
Commercial machine-vision illumination system	CCS ring light + controller + camera	Structured illumination imaging	1500 – 4000 €
Experimental PPGI setup (proposed illumination + camera)	FLIR Blackfly S BFS-U3-50S5M-C + proposed illumination	Multispectral PPGI acquisition	705 – 1105 €

In contrast, the proposed work focuses specifically on the multispectral illumination and control subsystem, which is implemented using off-the-shelf components at a total cost of approximately 105 €. For completeness, the total cost of the experimental setup used in this work can be estimated by combining the proposed illumination device with the monochromatic camera (FLIR Blackfly S BFS-U3-50S5M-C). This results in a total system cost of approximately 705–1105 €, depending on the camera configuration.

As shown in Table 1, this represents a reduction of one to two orders of magnitude in the cost of the illumination subsystem and a substantial reduction in total system cost compared to typical research-grade PPGI setups, while maintaining the functionality required for multispectral and synchronized measurements. The proposed system therefore provides an economically accessible platform for experimental PPGI research and educational applications.

• The system enables low-cost multispectral illumination for researchers developing camera-based physiological monitoring systems.
• It can be integrated into existing imaging setups to improve signal quality in photoplethysmography and related optical sensing applications.
• The modular and open-source design supports rapid prototyping of novel multispectral imaging experiments.
• The device can be adapted for studies involving tissue optics, perfusion mapping, or wavelength-dependent imaging.
• Its wireless control and configurable operation facilitate flexible laboratory automation and remote experimentation.

## 4 Design files summary

**Table:** 

Design file name	File type	Open source license	Location of the file
PCB_control_unit	CAD Files	CC BY 4.0	*https://data.mendeley.com/datasets/cj2bwfxrg4/2*
PCB_illumination_unit	CAD Files	CC BY 4.0	*https://data.mendeley.com/datasets/cj2bwfxrg4/2*
Protective_cover_top_bottom.stl	CAD Files	CC BY 4.0	*https://data.mendeley.com/datasets/cj2bwfxrg4/2*
Illumination_device_app.m	Matlab Files	CC BY 4.0	*https://data.mendeley.com/datasets/cj2bwfxrg4/2*
IlluminationDeviceAppC	Qt Creator files	CC BY 4.0	*https://data.mendeley.com/datasets/cj2bwfxrg4/2*
Illumination_device_firmware	Microchip studio Files	CC BY 4.0	*https://data.mendeley.com/datasets/cj2bwfxrg4/2*

• PCB_control_unit – Contains schematics and layout files for the control PCB integrating the microcontroller, communication interfaces, and LED driver control circuitry.
• PCB_illumination_unit – Provides the PCB design for the circular LED array enabling multispectral illumination with uniform spatial distribution.
• Protective_cover_top_bottom.stl – 3D-printable enclosure files designed to house and protect the complete device assembly.
• Illumination_device_app.m – MATLAB-based control application enabling real-time configuration of illumination modes, intensity, and frequency via serial communication.
• IlluminationDeviceAppC – Cross-platform Qt-based control software providing an open-source alternative to MATLAB for device operation.
• Illumination_device_firmware – Embedded firmware for the ATmega328P microcontroller implementing LED control, timing, and communication protocols.

## 5 Bill of materials summary

**Table:** 

Designator	Component	Pieces	Cost per unit −currency	Total cost − currency	Source of materials
PCB_device	PCB	5	0.4 €	2 €	https://jlcpcb.com/
PCB_illumination device	PCB	5	0.4 €	2 €	https://jlcpcb.com/
R1 – R2, R5 – R8	Resistor 100 kΩ	7	0.04657 €	0.32599 €	https://illuminationdevice.short.gy/P6ocNw
R3 – R4	Resistor 470 Ω	2	0.08554 €	0.25662 €	https://illuminationdevice.short.gy/lOD0Fs
R9 – R11	Resistor 22.5 kΩ	3	0.0901 €	0.2703 €	https://illuminationdevice.short.gy/zFwqLT
C1	Capacitor 1 µF	1	0.0781 €	0.0781 €	https://illuminationdevice.short.gy/aICPQq
C2	Capacitor 10 µF	1	0.1931 €	0.1931 €	https://illuminationdevice.short.gy/lmKTNM
C3 – C6	Capacitor 100 nF	4	0.04600 €	0.184 €	https://illuminationdevice.short.gy/itaCrp
JP1 – JP12, RESET, CAM	Male pin header	1	0.242 €	0.242 €	https://illuminationdevice.short.gy/OuprPi
LED1, LED34	THT LED diode, 3 mm or 5 mm	2	0.482 €	0.482 €	https://illuminationdevice.short.gy/OabHbI
U$1 – U$3	LED driver AL5871QT16E-13	3	1.08 €	3.24 €	https://illuminationdevice.short.gy/YreAsP
U1	Digital potentiometer MCP4331-104E/ST	1	1.90 €	1.90 €	https://illuminationdevice.short.gy/X9P7RY
U2	Microcontroller ATMEGA328P-AN	1	2.69 €	2.69 €	https://illuminationdevice.short.gy/BAnUoi
U3	LDO Voltage Regulator MIC29300-3.3 wt	1	3.37 €	3.37 €	https://illuminationdevice.short.gy/g3OzVc
U4	Bluetooth module BM78SPPS5MC2-0002AA	1	11.69 €	11.69 €	https://illuminationdevice.short.gy/2LfpMX
J1	Power supply connector, rated at min. 3A DC-005	1	0.254 €	0.254 €	https://illuminationdevice.short.gy/ATJmkv
LED1 – LED7 (illumination unit)	Green LED diode PM2E-3LGE-SD	7	1.26 €	8.82 €	https://illuminationdevice.short.gy/eojSZp
LED8 – LED19 (illumination unit)	Red LED diode PM2E-3LRE-SD	11	0.948 €	10.43 €	https://illuminationdevice.short.gy/ISXYV8
LED20 – LED35 (illumination unit)	NIR LED diode PM2E-2LJE-SD	15	2.16 €	32.4 €	https://illuminationdevice.short.gy/DfHuy6
3D printer filament	PLA Matte	1	23.76 €	23.76 €	https://overture3d.com/products/overture-matte-pla
**Total cost:**	105 €				

*All prices are exclusive of VAT and applicable duty charges.

## 6 Build instructions

The construction of the device involves the procurement of printed circuit boards (PCBs) for both the control unit and the illumination unit. Subsequently, both PCBs are assembled by mounting the components in accordance with the circuit schematics. After the assembly of all components on both PCBs, the firmware is uploaded to the ATmega328P microcontroller.

*Other necessary tools for assembly*

• Soldering iron
• Solder wire (Sn60Pb40 / lead-free)
• Multimeter
• Side cutters
• Atmel ICE Programmer (for ATmega328P) [20]

### 6.1 PCB assembly

For PCB fabrication, it is recommended to use experienced manufacturers capable of delivering high-quality printed circuit boards at reasonable cost and with short turnaround times, such as JLCPCB. The proposed device is composed of two functionally distinct subsystems: a control unit and an illumination unit. Consequently, both PCBs must be fabricated and assembled separately.

**Fig. 3:**
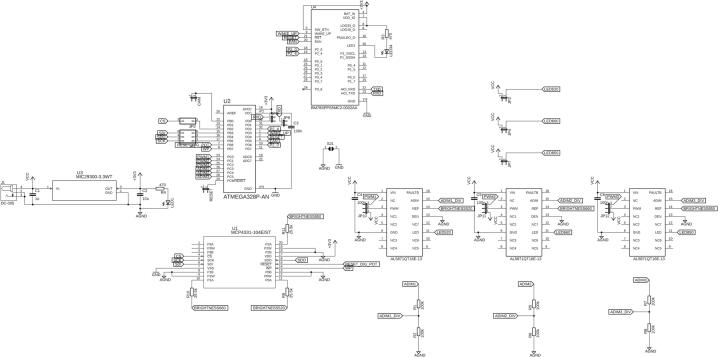
Device schematic created in EAGLE CAD Software.

**Fig. 4:**
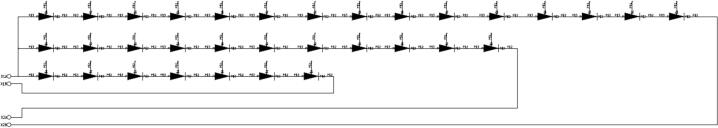
LED panel schematic created in KiCAD Software.

The complete circuit schematics of the system, including nominal component values and reference designators, are presented in Fig. 3. and Fig. 4. The control unit PCB was designed using Autodesk EAGLE, which provides robust support for digital and mixed-signal circuit layout. In contrast, the illumination unit PCB was developed using KiCad, as this design environment offers greater flexibility for routing and arranging components on irregular and circular board geometries, which are required for the LED array. This separation of design tools and hardware subsystems enables independent optimization of control logic and high-current illumination circuitry, while also simplifying future modifications and maintenance.

**Fig. 5:**
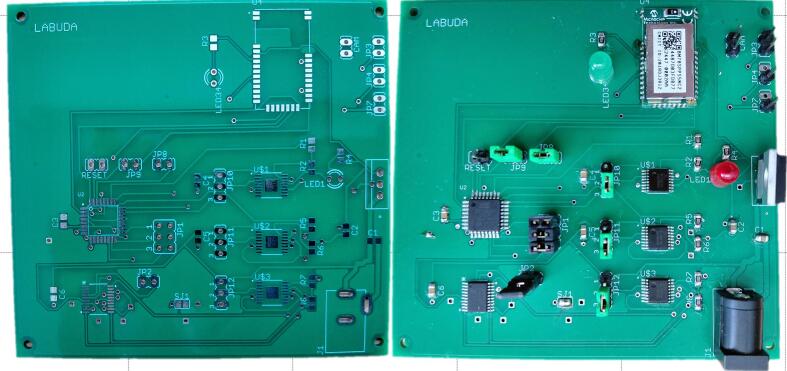
Control unit PCB before and after components assembly.

**Fig. 6:**
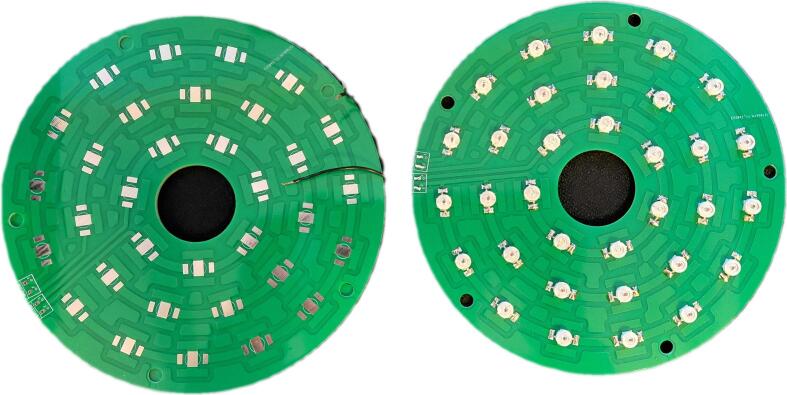
Illumination unit PCB before and after components assembly.

Fig. 5. and Fig. 6. present the physical realization of the proposed system, showing the control unit PCB and the illumination unit PCB both before and after component assembly. The unpopulated boards illustrate the routing of signal and power traces, via placement, and overall board layout, while the assembled versions demonstrate the final placement of integrated circuits, passive components, connectors, and LED elements.

The control unit PCB contains the microcontroller, power regulation circuitry, communication interfaces, and LED driver control components. In contrast, the illumination unit PCB features a circular layout populated with multiple high-power LEDs arranged in concentric rings, enabling uniform multi-wavelength illumination. The circular geometry of the illumination PCB is specifically designed to accommodate optical requirements and mechanical integration with the imaging system.

During the assembly process, careful soldering practices are essential to ensure reliable operation and long-term stability of the system. In particular, the soldering iron temperature should be properly controlled and maintained at approximately 330 °C. This temperature provides a suitable compromise between achieving high-quality solder joints and avoiding excessive thermal stress, which could otherwise lead to damage or reduced lifetime of sensitive components such as integrated circuits and LED diodes. Additionally, uniform soldering helps minimize contact resistance and improves thermal conduction, which is especially important for high-power LED applications.

**Fig. 7:**
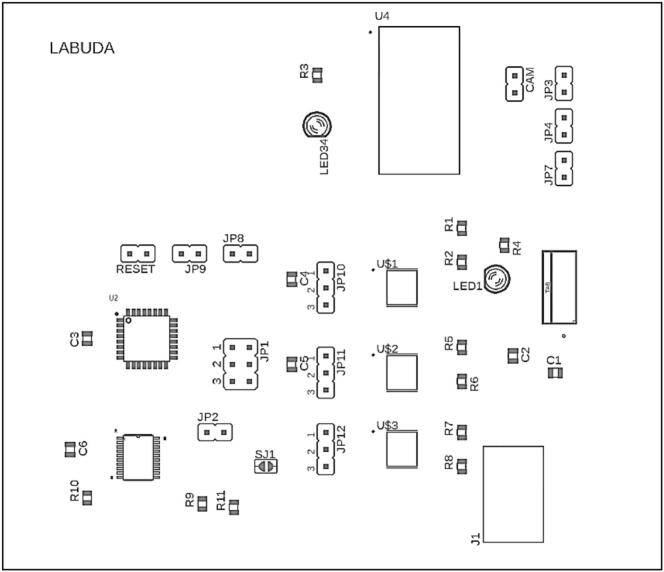
PCB Layout.

Description of individual jumpers (Fig. 7) and their functions:

• JP1, RESET – MCU programming pins via ISP (In-System Programming) (left column in order SCK, MISO, MOSI) & Short to enable SPI communication with digital potentiometer.
• JP3, JP4 and JP7 – Connection of individual LED strips (Green, Red, NIR). The top pin of the jumper is VCC for connecting the anode of the LED strips, and the bottom pin is the cathode for connection to the individual LED driver for each LED strip.
• JP10, JP11, JP12 – In position 3–2, enable PWM control of individual LED strips. Position 1–2 disable PWM control.
• JP2 – Enables SPI Slave select to control digital potentiometer.
• CAM – External trigger for camera frame acquisition. Use this jumper to connect up to two cameras via the top or bottom pin.
• JP8 and JP9 – The RX and TX pins enable programming and configuration of the Bluetooth module, including baud rate modification. Short to enable UART communication via Bluetooth module.

**Fig. 8:**
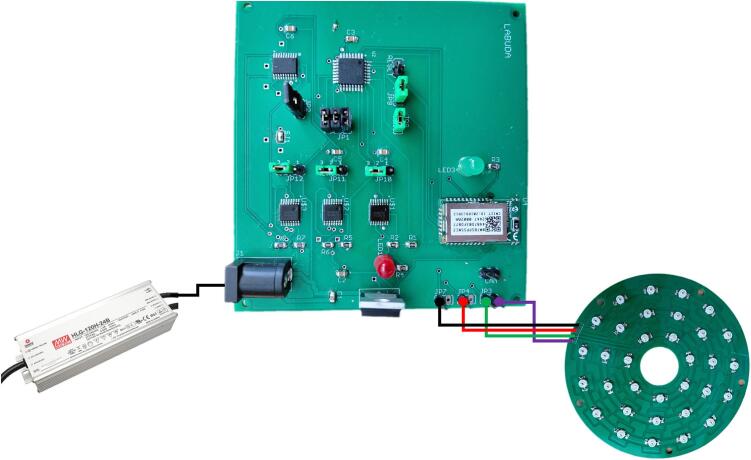
Block diagram illustrates the connections between the peripheral devices and the PCB.

Fig. 8 illustrates the electrical interconnection between the control unit, the illumination unit, and the external 24 V power supply. The power supply is connected directly to the control unit, which distributes the required voltage to the illumination circuitry and manages the switching of individual LED channels. The illumination unit is equipped with four electrical terminals. Three terminals are dedicated to the control of individual LED strings corresponding to the Green, Red, and Near-Infrared (NIR) wavelengths. The fourth terminal provides the power supply connection, which in the presented configuration operates at 24 V. Control signals generated by the control unit selectively activate the individual LED strings, enabling wavelength-specific illumination according to the selected operating mode. This connection scheme ensures a clear separation between power delivery and control signals, contributing to reliable operation and simplifying system integration and troubleshooting.

### 6.2 Uploading the firmware to the MCU

After assembling the printed circuit board and interconnecting all components, the operational firmware must be uploaded to the ATmega328P microcontroller. For this task, the freely available Microchip Studio development environment was used. The firmware is provided under the name illumination_device_firmware in the uploaded files. Microchip Studio is an integrated development environment (IDE) tailored for AVR and ARM microcontrollers produced by Microchip Technology. It supports code development, compilation, and debugging in C/C++, and allows direct programming of the target MCU using a compatible programmer. In this implementation, the Atmel-ICE debugger was employed as the programmer. Communication was established via the ISP interface using SPI signals. The correct pin configuration for connecting the Atmel-ICE to the PCB is listed in Table 2.

**Table 2:** Pinout for connecting the Atmel-ICE Debugger to PCB [20].

Atmel-ICE AVR ports pins	Target pins
Pin 1	SCK
Pin 2	GND
Pin 3	MISO
Pin 4	5 V
Pin 6	/RESET
Pin 9	MOSI

**Fig. 9:**
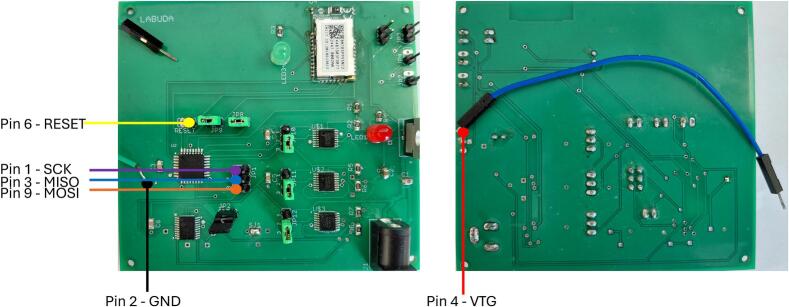
Visual representation of connection of Atmel-ICE Debugger to PCB*.*
(3) Connect the Atmel-ICE programmer to the power supply.
(4) Connect the power supply to control unit PCB.

Steps for establishing the connection between the programmer and the PCB:

(1) Remove jumpers JP1.
(2) Connect individual programmer pins according to Table 2. and Fig. 9. (red, black, blue, orange, purple, yellow wires).

Firmware Upload Procedure for the Control Unit:

Connect the Atmel-ICE programmer to both the computer and the PCB to be programmed, as shown in Fig. 9 and Table 2.

**Fig. 10:**
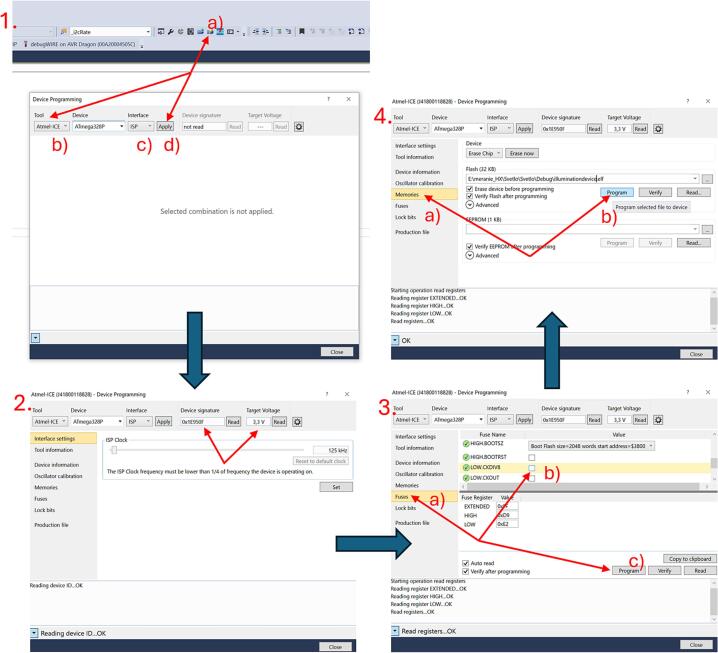
Detailed firmware upload instructions.
(2) Verify the connection by checking that the device signature (0x1E950F) and target voltage (3.3 V) are displayed (Fig. 10, step. 2.).
(3) Go to Fuses and disable the LOW.CKDIV8 fuse to set the MCU clock frequency to 8 MHz, then click Program (Fig. 10, step 3.).
(4) Finally, go to Memories and upload the compiled.hex file to the control unit by clicking Program (Fig. 10, step 4.).

(1) Open the project in Microchip Studio and click the Device Programming icon. Select Atmel-ICE as the programmer and ISP as the communication interface, then click Apply (Fig. 10, step 1.).

### 6.3 Design and print of the protective cover for the device

**Fig. 11:**
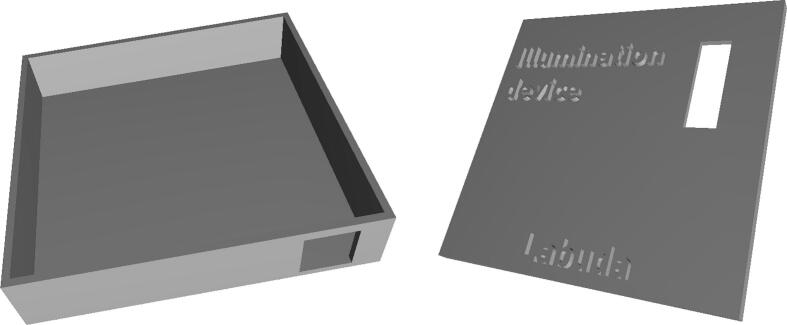
Protective cover designed in Autodesk Fusion 360.

To protect the device from external environmental influences and mechanical shocks, a custom protective enclosure was designed using Autodesk Fusion 360, as illustrated in Fig. 11. The enclosure was specifically tailored to accommodate the geometry of the control electronics while providing sufficient mechanical rigidity and protection during handling and operation.

The finalized enclosure design was manufactured using additive manufacturing, specifically 3D printing on a Bambu Lab A1 Mini printer. This manufacturing approach enables rapid prototyping, low production cost, and easy modification of the enclosure design if required. The enclosure is designed to fully house and protect all electronic components of the device while allowing access to connectors, indicators, and control elements.

The enclosure model is provided in STL format (cover.stl), which allows straightforward customization and further modification in Autodesk Fusion 360 to meet specific mechanical or application-related requirements. Both parts of the protective enclosure are first fabricated using a compatible 3D printer. After printing, all support structures are carefully removed to ensure proper fit and surface quality.

**Fig. 12:**
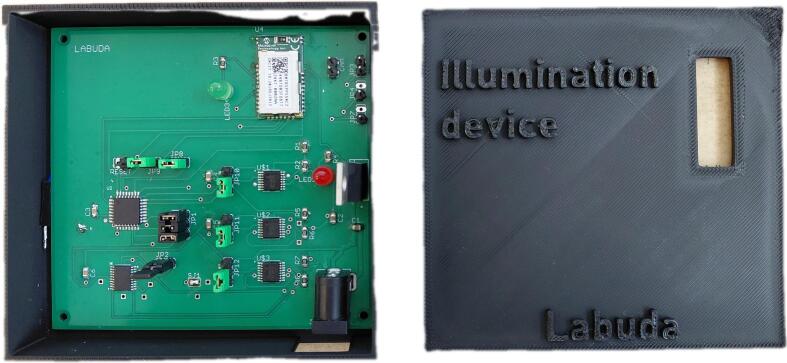
Placement of the device within the protective cover.

The fully assembled electronic device is then placed inside the enclosure according to the arrangement shown in Fig. 12, ensuring secure positioning and reliable mechanical integration. This enclosure solution provides effective protection for the device while maintaining ease of assembly and future adaptability.

## 7 Operation instructions

**Fig. 13:**
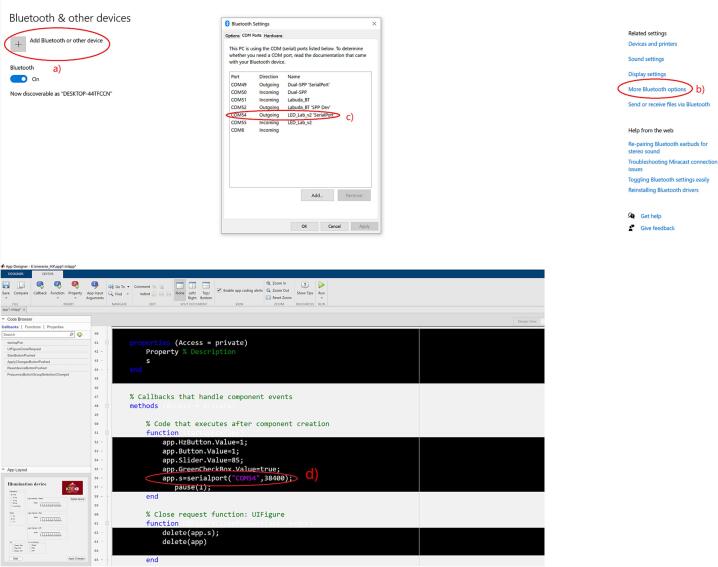
Identification of the newly assigned virtual COM port and its implementation in the application's source code.

After powering both the illumination unit and the control unit, the device is connected to the host computer via the Bluetooth communication interface. The connection procedure consists of the following steps and is illustrated in Fig. 13.

First, the device must be added and paired with the computer using the operating system’s Bluetooth settings, as shown in Fig. 13(a). Once Bluetooth is enabled, the illumination device appears in the list of available devices and can be paired in the standard manner.

After successful pairing, the operating system automatically assigns the device a virtual COM port. The assigned port number can be verified in the Bluetooth settings under the COM port configuration menu, as illustrated in Fig. 13(b) and 13(c). It is important to identify the outgoing COM port associated with the device, as this port is used for serial communication with the control software.

Finally, the control application implemented in MATLAB App Designer must be configured to use the correct COM port. As shown in Fig. 13(d), the assigned COM port number is entered directly into the application’s serial communication initialization code. Once the correct port is specified, the application can establish a reliable serial connection with the device, enabling real-time control of the illumination sequence and system parameters.

### 7.1 Device control application overview

**Fig. 14:**
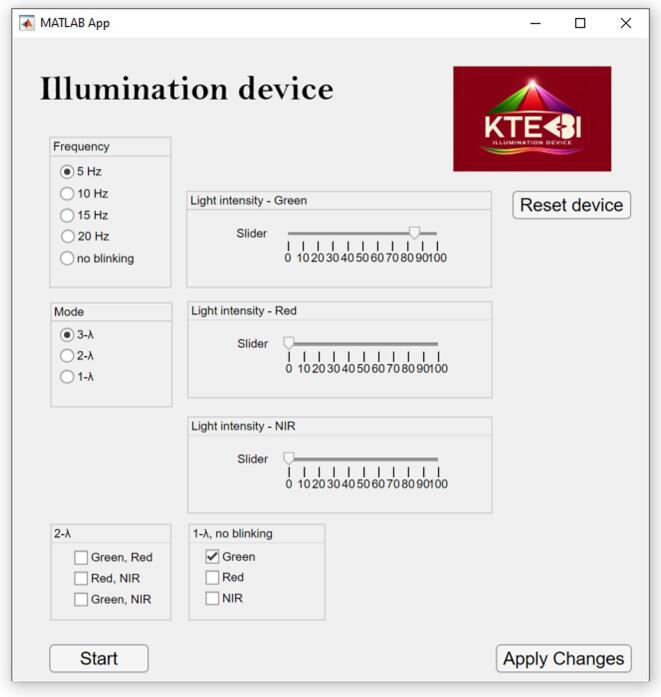
Graphical User Interface of the MATLAB Application.

Once the designed device is paired with the computer, the control application can be launched. Upon opening, the application automatically establishes a connection with the control unit. The user interface is shown in Fig. 14 and includes three main buttons: Start, Apply Changes, and Reset Device.

(1) Mode selection: Radio buttons labeled Mode allow setting the operating mode of the illumination unit: 3-λ mode: Sequential blinking of three strings of LEDs with different wavelengths (Green, Red, NIR). 2-λ mode: Sequential blinking of two strings of LEDs, with selectable combinations: Green + Red, Red + NIR, or Green + NIR. 1-λ mode: LEDs remain continuously lit according to the settings in “1-λ, no blinking.”
(2) Frequency: Radio buttons labeled Frequency allow easy adjustment of the blinking rate for 2-λ and 3-λ modes. Each LED string is activated sequentially, and all blink at the selected frequency.
(3) Light intensity: Sliders labeled Light Intensity Green, Red, NIR control the brightness of each LED pair from 0% to 100%.
(4) Applying settings: To confirm changes and send the configuration to the control unit, press Start or Apply Changes. The difference between the two is: Apply Changes: Settings are applied, but the LEDs do not start blinking. Start: Activates the illumination unit; LEDs blink or remain lit according to the current settings.
(5) Reset Device: Pressing this button restores the default device settings.

### 7.2 MATLAB application flowchart

**Fig. 15:**
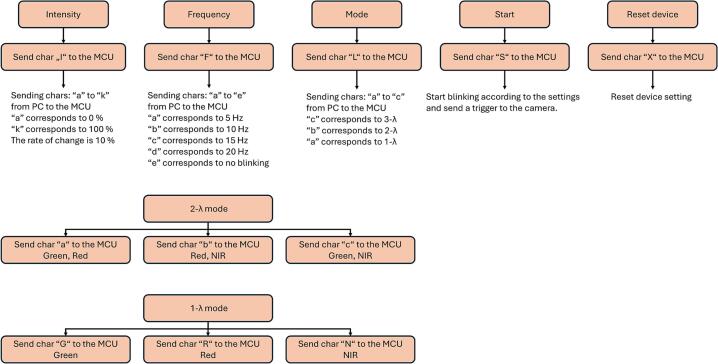
Designed MATLAB application command mapping interface.

Fig. 15 describes controlling an illumination system via communication with an MCU (microcontroller unit). The diagram shows five main control functions at the top level and additional settings for modes and wavelengths.

Top-Level Controls:

(1) Intensity – Sends the character “I” to the MCU to adjust the intensity.
(2) Frequency – Sends the character “F” to the MCU to adjust the frequency.
(3) Mode – Sends the character “L” to the MCU to set the device mode.
(4) Start – Sends the character “S” to the MCU to start the device.
(5) Reset device – Sends the character “X” to the MCU to reset the device.

Mode Selection:

• The device can operate in 3 modes: 3-λ mode, 2-λ mode and 1-λ mode.

3-λ mode (three-wavelength mode).

• Green & Red & NIR → Sends character “c” to MCU.

2-λ mode (two-wavelength mode).

• Green & Red → Sends character “a” to MCU.
• Red & NIR → Sends character “b” to MCU.
• Green & NIR → Sends character “c” to MCU.

1-λ mode (single-wavelength mode).

• Green → Sends character “G” to MCU.
• Red → Sends character “R” to MCU.
• NIR → Sends character “N” to MCU.

Flow Summary:

1. The user first selects the top-level control (Intensity, Frequency, Mode).
2. If Mode is selected, the user chooses between 2-λ mode or 1-λ mode.
3. Within the mode, the user selects the specific wavelength(s) to send the corresponding command character to the MCU.
4. The device starts by pressing Start button. The device is reset by pressing Reset device button. Changes in the settings are applied by pressing Apply Changes button.
5. Each selection communicates with the MCU via a single-character command, controlling the device behavior.

Essentially, the diagram is a command mapping interface: each button corresponds to a character sent to the MCU to control device intensity, frequency, mode, start/stop, reset, or wavelength settings.

Although a MATLAB-based application was developed for rapid prototyping and testing, the system is not dependent on proprietary software. An equivalent control application implemented in C++ using the open-source Qt framework is provided as part of the design files. Furthermore, the communication protocol is based on simple serial commands, allowing straightforward implementation using open-source tools such as Python (e.g., PySerial and PyQt). This ensures that the proposed system remains fully accessible and consistent with the low-cost and open-source design philosophy.

### 7.3 Microchip Studio firmware flowchart

**Fig. 16:**
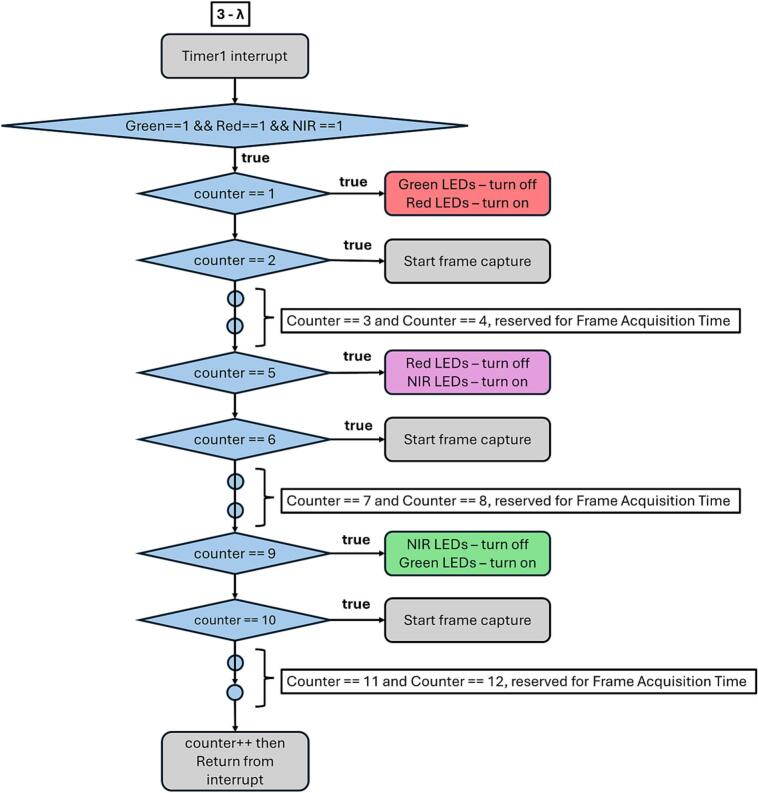
Microchip Studio firmware flowchart – 3 – λ, Timer Interrupt.

**Fig. 17:**
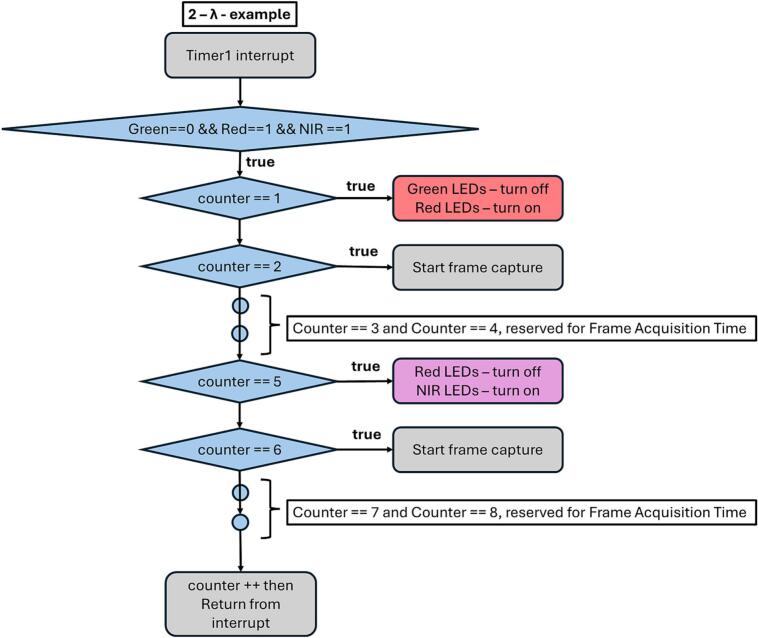
Microchip Studio firmware flowchart – 2– λ, Timer Interrupt.

The core part of the firmware that influences the blinking frequency of the LED strings and their switching is the interrupt generated by a 16-bit timer integrated in the MCU, as shown in Fig. 16. and Fig. 17. In the 3-λ mode, the interrupt occurs 12 times (Fig. 16). Each time the microcontroller enters the interrupt routine, either an LED string is switched or a trigger signal for the connected camera is activated, thereby initiating image acquisition. In the 2-λ mode, the interrupt occurs only 8 times, since only two LED strings are switched (Fig. 17). A sample calculation for a sampling frequency of 20 Hz can be defined for the 3-λ mode as follows:

(1) $$T_{T3\lambda }=\frac{\frac{F_{CPU}}{d}}{12F_{s}}=\frac{\frac{8MHz}{8}}{12∙20Hz}≅4167\mu s,$$

(2) $$T_{C3\lambda }=12∙4167\;\mu s=50ms,$$

where $F_{CPU}$ is frequency of CPU, d is prescaler of timer, $T_{T3\lambda }$ is period of one interrupt cycle and $T_{C3\lambda }$ is period of all interrupt cycles.

A sample calculation for a sampling frequency of 20 Hz can be defined for the 2-λ mode as follows:

(3) $$T_{T2\lambda }=\frac{\frac{F_{\mathrm{CPU}}}{d}}{8F_{s}}=\frac{\frac{8MHz}{8}}{8∙20Hz}=6250\mu s,$$

(4) $$T_{C2\lambda }=8∙6250\;\mu s=50ms,$$

where $F_{CPU}$ is frequency of CPU, *d* is prescaler of timer, $T_{T2\lambda }$ is period of one interrupt cycle and $T_{C2\lambda }$ is period of all interrupt cycles.

Fig. 16. illustrates the interrupt service routine (ISR) executed upon each interrupt generated by the MCU’s 16-bit timer. The routine controls both the switching of LED strings (Green, Red, and NIR) and the triggering of image acquisition by the connected camera. (For interpretation of the references to colour in this figure legend, the reader is referred to the web version of this article.)

At the beginning of the ISR, a check is performed to verify that all three LED strings (Green, Red, and NIR) are initially enabled. The subsequent behavior of the routine is governed by a software counter, whose value determines the specific action executed during a given interrupt.

When the counter equals 1, the Green LED string is turned off and the Red LED string is turned on. This change marks the transition to the Red illumination phase.

At the next interrupt, when the counter reaches 2, a trigger signal is generated to start frame capture by the camera under Red illumination.

As the counter continues to increment, no action is taken until the counter reaches 5. At this point, the Red LED string is turned off and the NIR LED string is turned on, switching the system to near-infrared illumination.When the counter equals 6, a new frame capture is initiated, corresponding to the NIR illumination state.

Later in the sequence, when the counter reaches 9, the NIR LED string is turned off and the Green LED string is turned on, returning the system to Green illumination.

At the following interrupt, when the counter equals 10, another camera trigger signal is generated to acquire an image under Green illumination.

After each interrupt execution, the counter is incremented, and the routine exits until the next timer interrupt occurs. This interrupt-driven sequence ensures precise synchronization between LED switching and camera triggering while maintaining a deterministic and repeatable timing pattern.

Fig. 17 depicts the operation of the timer-driven interrupt service routine (ISR) used in the 2-λ illumination mode, where only the Red and NIR LED strings are alternately activated. The ISR is invoked periodically by Timer1, which defines the timing of LED switching and camera triggering.

At the beginning of the ISR, a condition is evaluated to confirm the active illumination configuration, namely that the Green LED string is disabled, while the Red and NIR LED strings are enabled. Once this condition is satisfied, the subsequent actions are controlled by a software counter that increments on each interrupt occurrence.

When the counter reaches 1, the currently active Green LED string is turned off and the Red LED string is turned on, initiating the Red illumination phase. At the next interrupt, when the counter equals 2, a trigger signal is generated to start frame capture by the camera under Red illumination.

The counter continues to increment without further actions until it reaches 5. At this point, the Red LED string is turned off and the NIR LED string is turned on, switching the illumination to the near-infrared wavelength. When the counter equals 6, another camera trigger signal is issued, initiating image acquisition under NIR illumination.

After executing the corresponding action for the current counter value, the counter is incremented and the ISR exits. The routine then waits for the next Timer1 interrupt. This interrupt-driven sequence ensures deterministic timing and precise synchronization between LED switching and camera triggering while implementing a reduced switching cycle compared to the 3-λ mode.

**Fig. 18:**
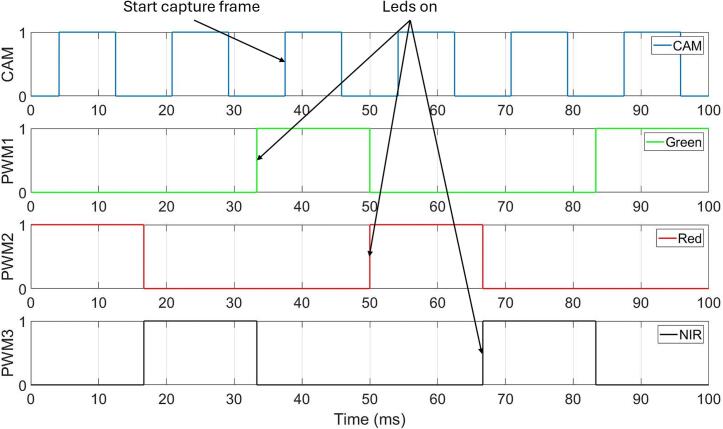
Timing diagram of proposed device.

Fig. 18. shows the timing relationships between the camera trigger signal and the PWM control signals for the Green, Red, and NIR LED strings in the 3-λ mode. (For interpretation of the references to colour in this figure legend, the reader is referred to the web version of this article.)

The top waveform (CAM) represents the camera trigger signal, which is asserted once per LED illumination interval to initiate image acquisition. The three lower waveforms (PWM1–PWM3) correspond to the PWM control signals driving the Green, Red, and NIR LED strings, respectively. At any given time, only one LED string is active, ensuring sequential multi-wavelength illumination.

The system operates with a sampling frequency of $F_{s}=20\;\text{Hz}$, resulting in a total cycle period $T_{c}=50\;\text{ms}$. Within this cycle, the illumination alternates between the Green, Red, and NIR LED strings. The time interval between switching two consecutive LED strings is:

(5) $$T=\frac{T_{c}}{3}=16.668\;\text{ms.}$$

As shown in the diagram, each LED string is first enabled via its PWM signal. After a short delay of 4.167 ms, the camera trigger signal is asserted. This delay allows the optical and electrical transients caused by LED switching to decay before image acquisition begins. The arrows in the Fig. 18. highlight this offset between the rising edge of each PWM signal and the corresponding camera trigger pulse.

This timing scheme ensures that each captured image corresponds to a stable illumination state, while maintaining precise synchronization between LED switching and camera triggering across all three wavelengths.

## 8 Validation and characterization

Adjusting the LEDs chain brightness via digital potentiometer.

In the proposed illumination system, high-power LEDs with peak wavelengths of 520 nm, 625 nm, and 855 nm were employed to enable multispectral operation. These wavelengths correspond to the green, red, and near-infrared regions of the electromagnetic spectrum, respectively, and allow flexible spectral configuration depending on the selected operating mode.

Because LEDs emitting at different wavelengths exhibit different forward voltage drops, and the sensing assembly is powered by a MEAN WELL HLG-120H-24 power supply providing a constant output voltage of 24 V, it was necessary to determine the appropriate number of LEDs connected in series for each wavelength. Owing to inaccuracies in the voltage specifications provided by the LED manufacturer, the forward voltage of the LEDs was experimentally determined under nominal operating conditions.

The measurements were performed using a laboratory-grade stabilized power supply with current-limiting capability. The current limit was set to 700 mA, corresponding to the rated operating current of the LEDs. Each LED was connected in the forward direction while the supply terminals were short-circuited to enable constant-current operation, and the resulting forward voltage drop was recorded. The maximum number of LEDs that could be connected in series was then calculated by dividing the available supply voltage (24 V) by the measured forward voltage. Finally, this value was rounded up to ensure that the current flowing through the LED string did not exceed the nominal 700 mA.

The number of LEDs (in one LED string) can therefore be calculated as follows:

(6) $$N_{\text{LED}}=\frac{V_{cc}}{V_{F}},$$

where $N_{\text{LED}}$ (−) represents the number of high-power LEDs in each series, $V_{cc}$ (V) corresponds to the supply voltage 24  V and $V_{F}$ (V) represents the measured voltage drop of a high-power LED in series.

From the quantum efficiency curve of the monochromatic camera BFS-U3-50S5M-C [21], it is evident that, among the three wavelengths used in this study, the camera exhibits the highest sensitivity at 520 nm and the lowest sensitivity at 855 nm. In the illumination design, this sensitivity variation is compensated by the selected number of high-power LEDs at each wavelength. Specifically, the fewest LEDs are used at 520 nm (7 LEDs), where the camera sensitivity is highest, while a moderate number is employed at 625 nm (11 LEDs). The greatest number of LEDs is allocated to 855 nm (15 LEDs), corresponding to the lowest quantum efficiency of the camera at this wavelength. Consequently, the calculated distribution of high-power LEDs is appropriate, as it effectively compensates for the camera’s quantum efficiency curve and ensures a more balanced detected signal across all wavelengths.

The maximum forward current of the LEDs is specified as 700 mA, which defines the upper safe operating limit for continuous operation. To ensure reliable performance and prevent overcurrent conditions, the LED driving circuitry was designed with a controlled current margin.

The maximum output current provided by the LED driver is 750 mA, allowing sufficient headroom for regulation while maintaining safe operating conditions for the LEDs. This margin ensures stable current control under varying thermal and electrical conditions without exceeding the maximum rated current of the LED devices.

It is important to note that each LED string is driven by an independent LED driver, which enables precise and individual current control for each spectral channel. This architecture improves system reliability, simplifies thermal management, and allows independent intensity adjustment of each LED wavelength.

The LED driver in technical documentation determines the forward current flowing through the LEDs according to the following relationship:

(7) $$I_{\text{LED}x}=12000\frac{1.5\;\text{V}}{R_{\text{REF}}+\left(R_{11}orR_{10}orR_{9}\right)},$$

where $I_{\text{LED}x}$ (A) is the LED current for a given channel, $R_{\text{REF}}$ (Ω) represents the effective resistance set by the digital potentiometer operating in rheostat mode, and $R_{11}$ (Ω) (NIR channel), $R_{10}$ (Ω) (Red channel), and $R_{9}$ (Ω) (Green channel) are fixed series resistors connected to the individual digital rheostats, Fig. 3.

The fixed resistors $R_{11}$, $R_{10}$, and $R_{9}$ were introduced to limit the maximum current to a safe value of 700 mA, corresponding to the maximum continuous current rating of the LEDs. This safety constraint is maintained even when the digital potentiometer is set to 0 or only wiper resistance 75 Ω, thereby preventing overcurrent conditions under all operating states.

**Fig. 19:**
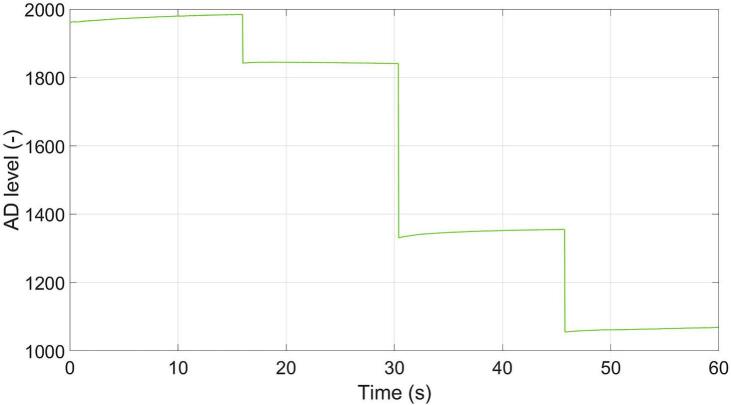
Example of brightness variation every 15  s using the digital potentiometer MCP43331. The brightness is controlled by wiper codes 0x00, 0x13, 0x5B, and 0x7F, corresponding to increasing resistance steps and resulting in four distinct brightness levels.

This current-limiting strategy ensures robust protection of the LEDs while preserving the full dynamic range of intensity control, Fig. 19.

(8) $$R_{\text{WA}}=100k\Omega -\frac{\mathrm{Code}\cdot 100k\Omega }{128}+75\Omega ,$$

where *R*_WA_ (Ω) is the resistance of the digital potentiometer between terminal A and wiper terminal W, Code is the 7-bit wiper register value (0–127), 75 Ω represents the typical wiper resistance.

Photobiological Safety Assessment of LED Ring Configurations According to IEC 62471.

Light-emitting diode systems operating in the visible and infrared spectral ranges must be evaluated for photobiological safety in accordance with IEC 62471:2006 (EN 62471). This standard defines exposure limits and classification into risk groups (RG0–RG3) based on potential hazards to the human eye and skin. This chapter presents a quantitative safety assessment of three LED configurations arranged in a ring geometry and using datasheet-based radiometric parameters, Table 3.

**Table 3:** Radiometric Parameters of the LEDs [16], [17].

LED Type	Ring Diameter (mm)	Irradiance @ 0.1 m (W·m^−2^)	Irradiance @ 0.65 m (W·m^−2^)
Green (PM2E-3LGE-SD)	66	58	1.37
Red (PM2E-3LRE-SD)	112	215	5.09
IR (PM2E-2LJE-SD)	158	349	8.27

The photobiological safety assessment must consider not only irradiance but also the geometrical extent of the source, expressed through the angular subtense α. The evaluated LED strings are arranged in circular rings with diameters of 66 mm, 112 mm, and 158 mm, respectively, resulting in large effective source diameters. At a distance of 0.1 m, the corresponding angular subtense values are significantly greater than 0.1 rad, placing all configurations within the extended source regime according to IEC 62471. In this regime, retinal hazards are evaluated using radiance limits that scale inversely with α, leading to substantially higher permissible exposure levels.

Despite the relatively high irradiance values observed for the visible LED configurations (approximately 58 W·m^−2^ for green and 215 W·m^−2^ for red), their large emitting area results in low radiance. Consequently, both retinal thermal and blue-light hazards remain well below the corresponding IEC 62471 limits. In contrast, the infrared configuration represents the most critical case, as the IEC 62471 infrared hazard is defined directly in terms of irradiance rather than radiance. The calculated irradiance of 349 W·m^−2^ at 0.1 m exceeds the Exempt Group limit of 100 W·m^−2^, indicating a potential hazard at close distances.

Therefore, while all configurations exhibit elevated irradiance at short distances, only the infrared system poses a photobiological risk under the analyzed conditions. The visible LED systems remain compliant with IEC 62471 due to their extended source geometry and correspondingly low radiance.

To support the safety assessment under practical operating conditions, the irradiance at the actual working distance of 65 cm was approximated using inverse-square distance scaling. Based on the measured irradiance values at 0.1 m, the corresponding irradiance at 65 cm is approximately 1.37 W·m^−2^ for the green channel, 5.09 W·m^−2^ for the red channel, and 8.27 W·m^−2^ for the infrared channel. These values are well below the IEC 62471 infrared exposure limit of 100 W·m^−2^. Therefore, at the intended operating distance, all illumination channels comply with the Exempt Group (RG0) classification, confirming safe operation during typical non-contact PPGI measurements.

It should be noted that all measurements presented in this article were performed at a fixed distance of 65 cm between the illumination device and the subject. This distance was selected to reflect typical non-contact PPGI measurement conditions.

Assessment of Spatial Illumination Uniformity in the Illumination Device.

The green illumination channel is implemented using a ring-based configuration of seven PM2E-3LGE-SD green LEDs, mounted on a circular printed circuit board PCB surrounding the camera aperture (Fig. 6). The LEDs are evenly distributed along the ring to provide quasi-symmetric illumination of the observed scene. The PCB layout (Fig. 6) and populated board confirm a uniform angular spacing and consistent orientation of the emitters.

**Fig. 20:**
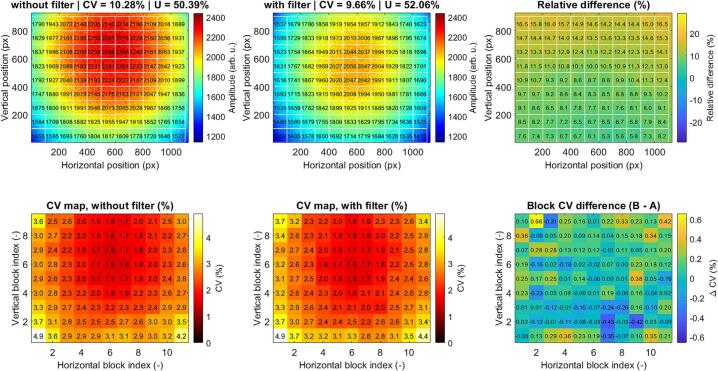
Spatial intensity distribution of the green illumination channel before (left) and after (center) application of a diffusion filter. The relative difference map (right) highlights redistribution of optical power, with reduced central intensity and increased peripheral illumination. Block-wise coefficient of variation (CV) maps (bottom row) demonstrate local intensity variability, while the ΔCV map indicates overall improvement in spatial uniformity after diffusion. (For interpretation of the references to colour in this figure legend, the reader is referred to the web version of this article.)

The spatial illumination distribution generated by this configuration was evaluated using block-wise averaging (100 × 100 pixels) of the captured green-channel intensity images. The measurements were performed using a matte white reference surface (standard 80 g/m^2^ office paper) positioned within the field of view to provide a uniform diffuse reflectance target. The resulting maps (Fig. 20, top row) reveal a characteristic radially symmetric intensity profile, with maximum values exceeding 2200 arb. u. in the central region and decreasing toward approximately 1450 arb. u. at the periphery in the baseline configuration (*without filter*).

After system modification using the Lee 216 white diffusion filter (*with filter*), the illumination becomes more evenly distributed, as evidenced by a reduction of peak central intensity and an increase in peripheral intensity. Quantitatively, the coefficient of variation (CV) decreases from 10.28% to 9.66%, while the global uniformity (min/max ratio) increases from 50.39% to 52.06%, indicating improved homogeneity.

The relative difference map shows a systematic redistribution of optical power, with a central intensity reduction of approximately 5–10% and a peripheral increase of approximately 10–16%, confirming that the modification effectively compensates for the inherent radial falloff of the ring illumination geometry.

Block-wise CV maps further indicate that spatial variability is lowest in the central region (∼1.6–2.0%) and increases toward the edges (∼3.0–4.9%). The CV difference map (ΔCV) demonstrates predominantly negative values in peripheral regions, confirming a reduction in local intensity variability after modification.

**Fig. 21:**
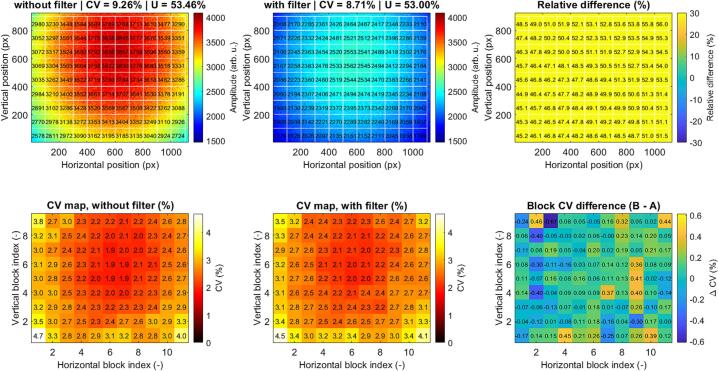
Spatial intensity distribution of the IR illumination channel before (left) and after (center) application of a diffusion filter. The relative difference map (right) highlights redistribution of optical power, with reduced central intensity and increased peripheral illumination. Block-wise coefficient of variation (CV) maps (bottom row) demonstrate local intensity variability, while the ΔCV map indicates overall improvement in spatial uniformity after diffusion.

The infrared illumination channel employs an outer ring configuration with 15 evenly distributed LEDs, providing increased angular coverage compared to the green channel. In the baseline configuration, the illumination exhibits a radially symmetric profile with peak intensities of approximately 3800–3900 arb. u. in the center, decreasing to 2600–3000 arb. u. toward the periphery (Fig. 21.). The corresponding CV and uniformity are 9.26% and 53.46%, respectively.

After applying the diffusion filter, the illumination becomes more uniform, with reduced central intensity and increased peripheral intensity. The CV decreases to 8.71% (−0.55%, ∼5.5% relative reduction), while uniformity remains stable at 53.00%. The relative difference map indicates a strong redistribution of optical power, with central attenuation of up to ∼ 50%. The ΔCV map confirms a consistent reduction in local variability, particularly in peripheral regions.

The red channel exhibited spatial characteristics closely matching those of the infrared channel in terms of uniformity and variability. Quantitative evaluation confirmed comparable CV and uniformity values. For brevity, only representative results for the green and infrared configurations are presented.

Employing a diffuser with higher optical density than Lee 216 is expected to further enhance illumination homogeneity by mitigating residual spatial gradients; however, this improvement would occur at the expense of reduced radiant intensity, as stronger diffusion inherently introduces additional scattering losses.

Thermal Behavior and Heat Dissipation Analysis of the Illumination Device.

**Fig. 22:**
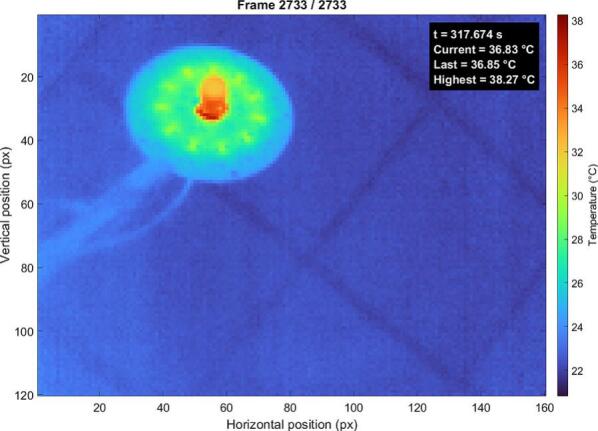
Thermal map of the red LEDs under continuous operation. (For interpretation of the references to colour in this figure legend, the reader is referred to the web version of this article.)

The thermal performance of the illumination device was evaluated using infrared thermography. All measurements presented in this section were conducted at a fixed distance of 65 cm between the illumination system and the observed area. The measurements were performed under steady-state conditions after thermal stabilization of the LEDs. The illumination system includes a central opening accommodating a BFS-U3-50S5M-C camera, which contributes additional heat to the overall thermal profile. This effect is most pronounced in the green LED channel, as these LEDs are positioned closest to the central aperture, where heat generated by the camera is concentrated, leading to increased local thermal loading. Each LED string is designed with dedicated thermal pads located directly beneath the LEDs, ensuring effective heat transfer from the junction to the PCB and improving overall thermal management of the system. Thermal images of the three LED configurations (red, infrared, and green) reveal distinct temperature distributions corresponding to their electrical power and optical characteristics. The red LED configuration (Fig. 22.) exhibits a moderate and relatively uniform temperature distribution, with a maximum temperature of approximately 38.3 °C. The central region shows slightly elevated temperatures, influenced by both the LED board and the thermal contribution of the centrally located camera.

**Fig. 23:**
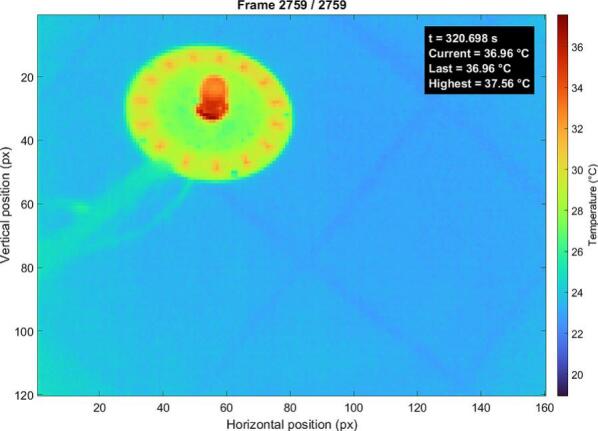
Thermal map of the IR LEDs under continuous operation.

The infrared LED configuration (Fig. 23.) demonstrates similar thermal behavior, with a maximum temperature of approximately 37.6 °C.

**Fig. 24:**
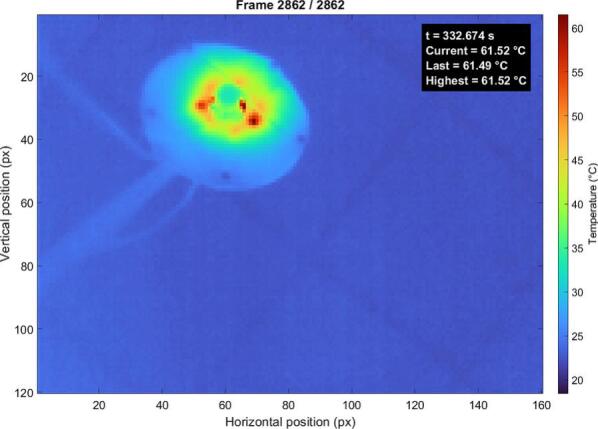
Thermal map of the green LEDs under continuous operation. (For interpretation of the references to colour in this figure legend, the reader is referred to the web version of this article.)

The temperature distribution is relatively homogeneous, indicating efficient heat spreading and lower overall thermal load. The green LED configuration (Fig. 24.) shows the highest thermal loading, with peak temperatures reaching approximately 61.5 °C. The temperature distribution is less uniform, with visible hotspots corresponding to individual LEDs, indicating higher power dissipation and increased thermal stress. This increased temperature is attributed not only to the proximity of the green LEDs to the central aperture and the associated thermal contribution of the camera, but also to the higher local power density of the green LED configuration, resulting in increased heat generation and more localized thermal accumulation.

It should be noted that the thermal analysis presented above was performed under conditions of continuous operation at maximum brightness, without any modulation of the LED output. Under these conditions, the measured temperatures represent the worst-case thermal scenario for each configuration.

In practical applications, the illumination system may operate in a pulsed (blinking) mode with modulation frequencies such as 5 Hz, 10 Hz, 15 Hz, or 20 Hz. In such cases, the LEDs are not continuously driven at full power, resulting in a reduced average electrical input power and consequently lower heat generation.

Consequently, operation in pulsed mode is expected to reduce the maximum temperature of the LEDs, improve thermal uniformity, decrease thermal stress and aging effects.

This effect is particularly beneficial for the green LED configuration, which exhibited the highest temperature under continuous operation. The use of pulsed driving can therefore serve as an effective strategy for thermal management without requiring additional hardware modifications.

Photoplethysmography imaging setup and test measurements.

**Fig. 25:**
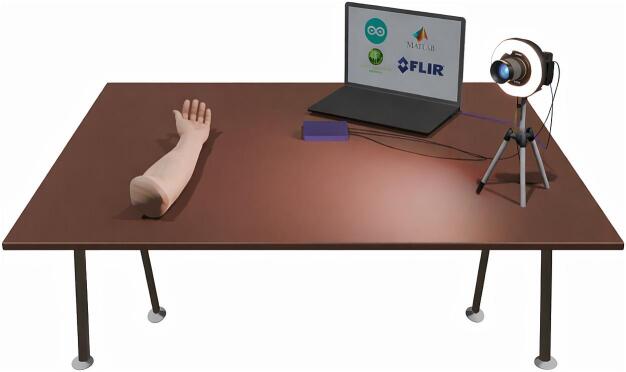
3D representation illustrating the measurement setup configuration.

To verify the functionality of the proposed photoplethysmography imaging illumination device, a series of measurements were performed using the experimental setup shown in Fig. 25. A key component of every PPGI measurement system is the imaging sensor, in the proposed setup, a monochromatic camera (BFS-U3-50S5M-C) was employed to avoid spectral cross-talk and to ensure wavelength-independent sensor response. The sampling frequency during the measurements was set to 20 FPS per channel (R, G, NIR). Therefore, the camera recorded at a frequency of 60 FPS.

As a light source, a designed multispectral illumination device was used. In addition to providing illumination, the device also controls the camera operation by triggering image acquisition in a synchronized manner. In the 3-λ acquisition mode, each consecutive image frame is captured under illumination at a different wavelength, specifically 520 nm, 625 nm, and 855 nm, enabling sequential multispectral PPGI measurements with a single monochromatic camera.

The illumination device features a ring-shaped geometry with a central aperture that allows direct mounting onto the camera lens, ensuring uniform illumination of the region of interest and a fixed geometric relationship between the camera and the light source. This configuration minimizes shadowing and improves measurement repeatability.

The measurement setup further includes a laptop computer for system control and data storage. During the experiment, the subject’s arm was placed on a foam pad to reduce motion artifacts and to maintain a stable measurement position. To enhance the pulsatile component of the PPGI signal, a warming and vasodilatory cream (Finalgon) was applied to the measurement area prior to data acquisition. The application of Finalgon increases local blood perfusion, thereby improving the signal-to-noise ratio of the recorded PPGI signals across all illumination wavelengths.

In photoplethysmography imaging measurements, the use of a diffusion filter on the illumination device is essential to ensure spatially uniform and soft illumination across the region of interest. In the proposed system, a LEE 216 white diffusion filter was applied to the multispectral illumination device to suppress direct, highly directional light emitted by individual LEDs.

Without diffusion, the illumination pattern is dominated by localized intensity maxima (hotspots) corresponding to the LED positions, which leads to non-uniform illumination, increased specular reflections, and spatially varying signal amplitudes. These effects can introduce artifacts into the extracted PPGI signals and negatively affect the accuracy and repeatability of blood volume pulse estimation.

The diffusion filter scatters the emitted light, producing a homogeneous illumination field that minimizes intensity gradients, shadows, and surface reflections. This results in more consistent pixel-wise signal amplitudes, improved robustness of region-of-interest selection, and an overall enhancement of the signal-to-noise ratio of the measured PPGI signals.

**Fig. 26:**
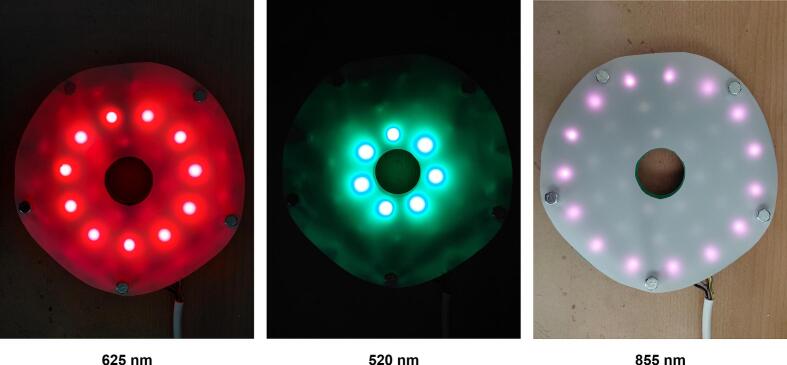
Illumination unit equipped with a Lee 216 White Diffusion filter, shown with different light sources activated.

Fig. 26. illustrates the multispectral illumination device equipped with the diffusion filter under illumination at 625 nm, 520 nm, and 855 nm. Despite the wavelength-dependent emission characteristics of the LEDs, the diffusion filter ensures a comparable spatial light distribution across all spectral channels, thereby supporting reliable multispectral PPGI acquisition.

**Fig. 27:**
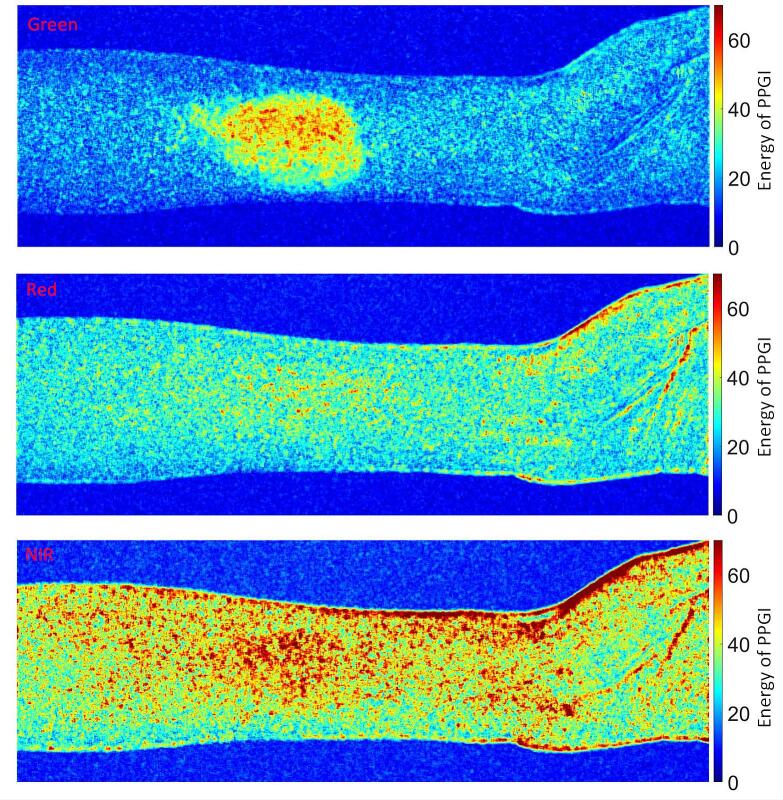
Spatial Distribution of Perfusion Energy Using Green, Red, and Near-Infrared Illumination. (For interpretation of the references to colour in this figure legend, the reader is referred to the web version of this article.)

The comparison in Fig. 27. reveals pronounced wavelength-dependent differences in both the magnitude and spatial specificity of the pulsatile energy distribution following topical vasodilation. Illumination at 520 nm yields a highly localized region of elevated pulsatile energy, corresponding to the Finalgon application site, consistent with the strong superficial sensitivity of green light to microvascular perfusion changes. In contrast, the corresponding red and near-infrared maps exhibit only weak residual traces of this localized hotspot, indicating reduced spatial specificity of surface-localized perfusion changes at longer wavelengths.

At the same time, the near-infrared map displays a higher overall pulsatile energy distributed over a larger tissue area compared to the green and red channels. This observation is consistent with the larger effective sampling volume and reduced optical attenuation at longer wavelengths, leading to increased integration of pulsatile blood volume changes over deeper and spatially extended tissue compartments. Consequently, near-infrared illumination provides higher global pulsatile energy but lower spatial contrast for localized superficial perfusion changes.

Taken together, these results illustrate the complementary nature of multispectral PPGI: shorter wavelengths emphasize spatially localized superficial microvascular responses, whereas longer wavelengths capture more spatially averaged pulsatile activity from deeper tissue volumes. This wavelength-dependent trade-off between spatial specificity and effective sampling depth motivates the use of multispectral illumination for robust and physiologically informative PPGI measurements.

**Fig. 28:**
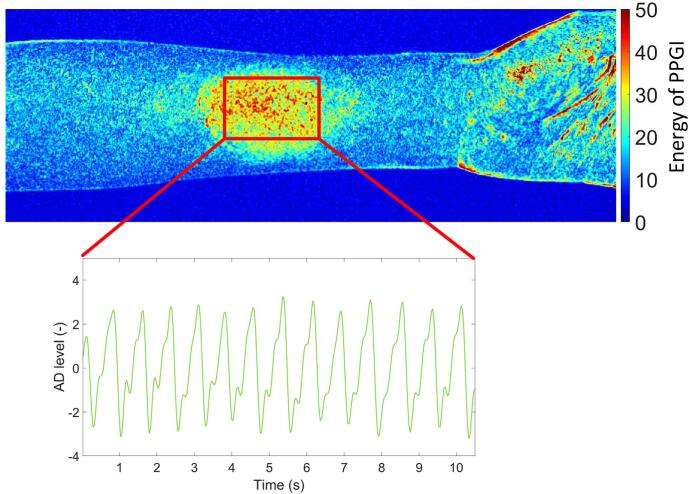
Green-Channel PPGI with Extracted Pulse Signal from ROI.

Test measurement obtained from the green color channel after application of Finalgon is shown in Fig. 28. The upper panel shows a spatio-temporal map of the PPGI signal energy, where warmer colors indicate higher pulsatile energy. The red rectangle denotes the selected region of interest (ROI) exhibiting the strongest pulsatile component. The lower panel presents the corresponding green-channel PPGI waveform extracted from the ROI. Following Finalgon application, the signal demonstrates a clear and regular pulsatile pattern with improved amplitude and signal-to-noise ratio, enabling reliable identification of cardiac cycles over the 10 s recording interval. The detailed process of creating perfusion maps and the algorithm can be found in [22].

SNR-Based Performance Comparison of Conventional PPGI, Contact Finger PPG, and the Proposed System.

Signal-to-noise ratio is a fundamental metric for evaluating the quality of physiological signals, as it quantifies the ratio of useful signal power to noise power. In PPGI, SNR plays a critical role in assessing system performance and ensuring reliable extraction of cardiovascular information.

**Fig. 29:**
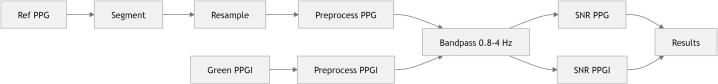
Flowchart of the signal-to-noise ratio estimation algorithm.

The proposed algorithm for SNR estimation is illustrated in Fig. 29. The method separates the pulsatile (signal) and non-pulsatile (noise) components of the PPGI signal. First, the reference contact PPG signal is resampled to match the sampling rate of the PPGI signal. Subsequently, both signals are mean-centered, detrended, and normalized to remove baseline drift and amplitude variations. A bandpass filter with a passband of 0.8–4 Hz is applied to isolate the physiological pulsatile component associated with cardiac activity. The filtered signal is considered the signal component, while the residual between the preprocessed signal and its filtered version is treated as noise. The SNR is then computed as the logarithmic ratio of signal power to noise power.

**Fig. 30:**
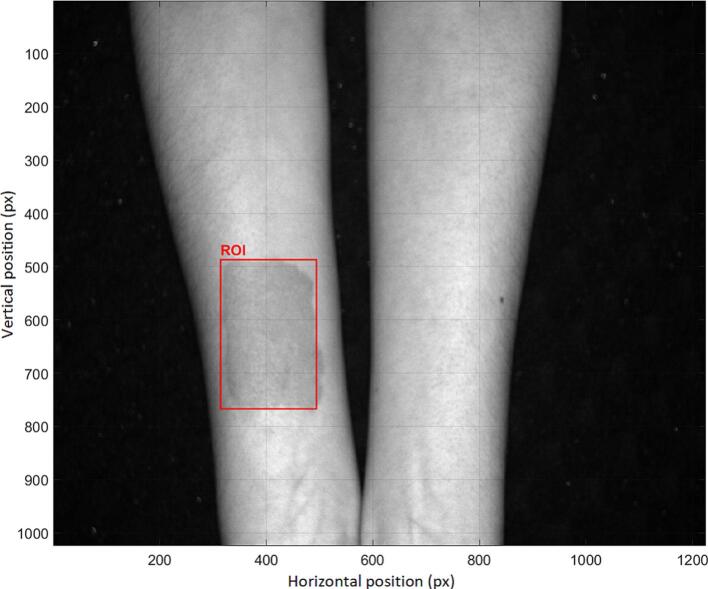
Region of Interest Selection for SNR Evaluation in PPGI Systems.

For quantitative evaluation, a region of interest ROI was defined, as shown in Fig. 30. The red rectangle indicates the ROI used for SNR computation in both the proposed system and the conventional PPGI approach. The monochromatic camera (BFS-U3-50S5M-C) operated at a resolution of 1224 × 1024 pixels, whereas the RGB camera (BFS-U3-32S4C-C) had a resolution of 1024 × 768 pixels. In both configurations, the gain was set to 5 and the exposure time to 5 ms.

The reference contact PPG signal, acquired from the finger using the Biopac MP36 system, achieved an SNR of 10.10 dB. The PPGI signal obtained using the monochrome camera with the proposed illumination device yielded an SNR of 4.81 dB. In comparison, the PPGI signal extracted from the green channel of the RGB camera under white-light illumination (Redhead RGB-60) achieved an SNR of 4.64 dB.

It should be noted that these SNR values were obtained from a single measurement on one subject under controlled laboratory conditions. Therefore, the 0.17 dB difference between the proposed system and the RGB-based reference should not be interpreted as a statistically significant performance difference.

**Fig. 31:**
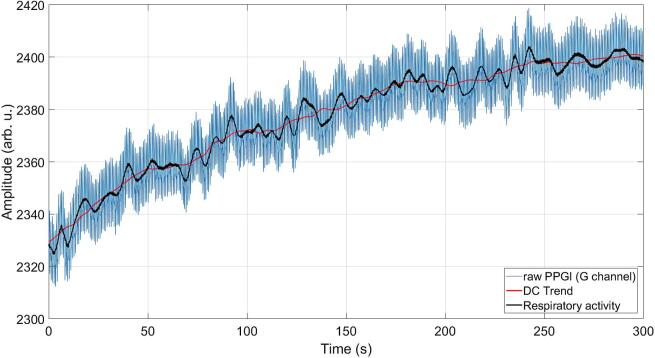
Example of a PPGI signal acquired under green illumination with DC drift and respiratory component.

As shown in Fig. 31, the recorded PPGI signal exhibits a gradual DC drift over time, which is primarily attributed to LED self-heating and the resulting change in emitted optical power. In addition to the high-frequency pulsatile component associated with cardiac activity, a low-frequency modulation is also present, corresponding to respiratory activity. The DC trend and respiratory component were extracted using appropriate filtering techniques to illustrate their contribution to the overall signal. After approximately 5 min of operation, the DC drift tends to stabilize, suggesting that the LED temperature approaches a steady-state condition.

## 9 Discussion

The presented results confirm that the proposed multispectral illumination system provides stable and sufficiently uniform optical conditions for photoplethysmography imaging. The spatial intensity measurements demonstrate that the ring-based LED configuration produces a radially symmetric illumination profile, which can be further improved using a diffusion filter. The observed reduction in coefficient of variation and increase in global uniformity indicate that the system is capable of delivering illumination conditions suitable for reliable extraction of pulsatile signals.

The multispectral design enables wavelength-dependent sensitivity to different tissue depths, as demonstrated by the perfusion energy maps. Green illumination emphasizes localized superficial perfusion changes, whereas near-infrared illumination provides higher overall pulsatile energy distributed across a larger tissue volume. This behavior is consistent with the known optical properties of biological tissue and confirms that the system can capture complementary physiological information across wavelengths. Such capability is particularly advantageous for improving robustness of PPGI measurements under varying conditions, including differences in skin tone, perfusion, and motion artifacts.

The signal-to-noise ratio analysis shows that the proposed system achieves performance comparable to conventional RGB-based PPGI under white-light illumination. Although the absolute SNR difference is small, the key advantage of the proposed system lies in its ability to independently control wavelength selection and illumination intensity. This flexibility enables optimization of measurement conditions for specific applications and provides a platform for investigating wavelength-dependent physiological effects.

Thermal characterization demonstrates that the system operates within acceptable temperature limits under continuous operation, with the highest thermal load observed in the green LED configuration. The results further indicate that pulsed operation can effectively reduce thermal stress and improve thermal stability without requiring additional hardware modifications. This is particularly relevant for long-duration measurements and contributes to improved system reliability.

From a design perspective, the use of linear LED drivers represents a deliberate trade-off between efficiency and signal quality. While switching drivers offer higher efficiency, linear drivers were selected to minimize electrical noise and eliminate switching artifacts that could degrade the PPGI signal. This choice prioritizes signal integrity, which is critical in optical biosensing applications where small intensity variations must be accurately detected.

The presented system is intended as a low-cost multispectral illumination and control platform for PPGI. It should be noted that the current validation is limited to a small number of measurements conducted under controlled laboratory conditions. Future work will therefore focus on extended validation across a larger population, evaluation under varying environmental conditions, and integration with real-time signal processing algorithms.

It should be noted that the open-source hardware and software design is not presented as a standalone scientific novelty, but rather as an enabling contribution that improves reproducibility, transparency, and accessibility of the proposed system.

## 10 Conclusion

This paper presented a low-cost three-wavelength illumination system for photoplethysmography imaging (PPGI), integrating independently controlled green, red, and near-infrared LED channels with synchronized operation and optional wireless control. The modular and open-source design enables straightforward replication and adaptation for research applications.

Experimental validation demonstrated that the system provides sufficiently uniform illumination, stable operation, and reliable extraction of pulsatile signals. Quantitative analysis confirmed that the performance of the proposed system is comparable to conventional PPGI approaches, while offering additional flexibility through multispectral control.

The results highlight the potential of the proposed device as an accessible and adaptable platform for non-contact physiological monitoring and for investigating wavelength-dependent effects in PPGI systems.

## Ethics statements

The authors have nothing to declare under this heading.

## CRediT authorship contribution statement

**Michal Labuda:** Writing – review & editing, Writing – original draft, Validation, Supervision, Software, Methodology, Formal analysis, Data curation, Conceptualization. **Ivan Kuchta:** Methodology. **Veronika Wohlmuthova:** Visualization, Software. **Jan Seleng:** Formal analysis, Data curation.

## Declaration of competing interest

The authors declare that they have no known competing financial interests or personal relationships that could have appeared to influence the work reported in this paper.
